# Synthesis of Aluminum Complexes Bearing 8-Anilide-5,6,7-trihydroquinoline Ligands: Highly Active Catalyst Precursors for Ring-Opening Polymerization of Cyclic Esters

**DOI:** 10.3390/polym9030083

**Published:** 2017-03-01

**Authors:** Shaofeng Liu, Jie Zhang, Weiwei Zuo, Wenjuan Zhang, Wen-Hua Sun, Hongqi Ye, Zhibo Li

**Affiliations:** 1 State Key Laboratory for Modification of Chemical Fibers and Polymer Materials, Donghua University, Shanghai 201620, China; zuoweiwei@iccas.ac.cn; 2 School of Polymer Science and Engineering, Qingdao University of Science and Technology, Qingdao 266042, China; zbli@qust.edu.cn; 3 Department of Chemistry and Engineering, Central South University, Changsha 410083, China; csuzhangj@163.com (J.Z.); yeslab@csu.edu.cn (H.Y.); 4 Key Laboratory of Engineering Plastics and Beijing National Laboratory for Molecular Sciences, Institute of Chemistry, Chinese Academy of Sciences, Beijing 100190, China; zhangwj@iccas.ac.cn

**Keywords:** aluminum complexes, crystal structures, ring-opening polymerization, biodegradable polyesters

## Abstract

The stoichiometric reactions of 8-(2,6-R^1^-4-R^2^-anilide)-5,6,7-trihydroquinoline (**LH**) with AlR_3_ (R = Me or Et) afforded the aluminum complexes **LAlR_2_** (**Al1**–**Al5**,**Al1**: R^1^ = *^i^*Pr, R^2^ = H, R = Me; **Al2**: R^1^ = Me, R^2^ = H, R = Me; **Al3**: R^1^ = H, R^2^ = H, R = Me; **Al4**: R^1^ = Me, R^2^ = Me, R = Me; **Al5**: R^1^ = Me, R^2^ = Me, R = Et) in high yields. All aluminum complexes were characterized by NMR spectroscopy and elemental analysis. The molecular structures of complexes **Al4** and **Al5** were determined by single-crystal X-ray diffractions and revealed a distorted tetrahedral geometry at aluminum. In the presence of BnOH, complexes **Al1**–**Al5** efficiently initiated the ring-opening homopolymerization of *ε*-caprolactone (*ε*-CL) and *rac*-lactide (*rac*-LA), respectively, in a living/controlled manner.

## 1. Introduction

Polyesters including polycaprolactone (PCL), polylactide (PLA), and their copolymers are ubiquitous engineering materials in our daily life and have attracted considerable attention over the past decades due to their potential as renewable resources and their biodegradable characteristics [1,2,3]. It is notable that they are not only biodegradable but also bioassimilable, and much interest has been focused on their biomedical and pharmaceutical applications such as drug delivery excipients, adsorbable surgical sutures, bone screws, and materials for tissue engineering [4].

A particularly convenient method for the synthesis of polyesters is the ring-opening polymerization (ROP) of cyclic esters using metal complexes as catalysts or initiators, including aluminum [5,6,7,8,9], rare earth metals [10,11,12,13], titanium and zirconium [14,15], magnesium and zinc [16,17,18,19,20,21,22], tin [23,24], and iron [25,26,27,28] complexes. Among these, aluminum complexes bearing ancillary ligands have attract the most of attention and are one kind of the most promising catalysts for ROP of cyclic esters owing to tremendous catalytic activities, low toxicity, excellent controllability over the molar mass, dispersities, and regio- or stereo-selectivities of the resultant polymers. The ancillary ligands in the aluminum-based catalytic systems have been proved to be an important role in determining the catalytic performances by tuning the electronic and steric properties. Gibson [29] systematically studied the factors influencing the ROP of *rac*-LA by (salen)Al complexes, for instance, which are well known as highly efficient catalysts, and found that high activities were favored by electron-withdrawing substituents on the phenoxy, but suppressed by large *ortho*-phenoxy substituents. In contrast, the isoselectivity was favored by sterically demanding *ortho*-phenoxy groups. More recently, Nomura [30] reported successful controlled random copolymerization of ε-CL and LA with a homo salen-Al catalyst by introduction of a bulky *^i^*Pr_3_Si group, which could narrow the reactivity ratio gap of ε-CL and LA.

**Scheme 1:**
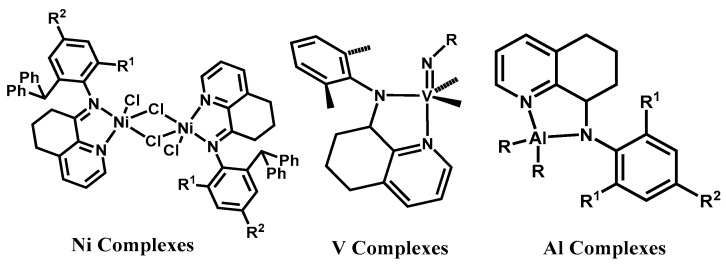
Highly active catalytic systems containing 5,6,7-trihydroquinolyl ligands.

Over the past few years, we studied Al complexes bearing bidentate and tridentate ligands such as bis-phenolate [31], 8-quinolinolates [32], aldiminophenolates [33], imidazolylphenolates [34] and amidates [35]. During the course of this research, it is clearly that thoughtfully tuning of the environments of the ligands, namely, incorporation of different substituents or heteroatoms in the framework of the ligands, could tremendously influence the observed catalytic activities and resulting properties of the products. Therefore, we continue to pursue the new catalytic models design. Recently, nickel complexes (Scheme 1, Left) containing 8-arylimino-5,6,7-trihydroquinolyl ligand [36,37,38,39] and vanadium complexes (Scheme 1, Middle) bearing 8-(2,6-dimethylanilide)-5,6,7-trihydroquinoline ligand [40] were reported exhibiting remarkable reactivity with ethylene, and the fused six-member-ring seemed to be the key fact in regard to the catalyst design. In this context, the Al complexes (Scheme 1, Right) bearing a series of 8-(2,6-R^1^-4-R^2^-anilide)-5,6,7-trihydroquinoline ligands have been prepared and applied as initiates for ring-opening homo and copolymerization of *ε*-CL and *rac*-LA.

## 2. Materials and Methods

### 2.1. General Considerations

Schlenk techniques or glove-box techniques were employed for compounds and reactions which are moisture/oxygen sensitive. *n*-Hexane, toluene and THF were dried by refluxing over sodium/benzophenone. CH_2_Cl_2_ was dried over CaH_2_, distilled and stored with activated molecular sieves (4A) under nitrogen. CDCl_3_ dried over CaH_2_ and C_6_D_6_ dried over Na/K were vacuum transferred prior to use. AlMe_3_ and AlEt_3_ were purchased from Aldrich. ε-CL was purchased from Aladdin and dried over CaH_2_. *rac*-LA was purchased from TCI and used as received. FT-IR and elemental analysis were performed on the Bruker Tensor 27 (Bruker, Qingdao, China) and Perkin-Elmer 2400II (PerkinElmer, Qingdao, China), respectively. NMR spectra were recorded on Bruker DMX-500 (^1^H: 500 MHz, ^13^C: 125 MHz, Bruker, Qingdao, China). The GPC analysis was carried out at 40 °C on Wyatt OPTILAB rEX with StyragelP8512-10E3A10 (the effective molar mass range is from 100 to 40,000, Wyatt Technology Corporation, Qingdao, China) using THF as the eluent. Molar mass and dispersity Ð were calculated using polystyrenes as standard, correcting factors of 0.56 and 0.58 for PCL and PLA, respectively [41].

### 2.2. Synthesis of 8-(2,6-R^1^-4-R^2^-anilide)-5,6,7-trihydroquinoline (**LH1**–**LH4**)

*Synthesis of 8-(2,6-^i^Pr-anilide)-5,6,7-trihydroquinoline (**LH1**).* The synthetic procedure of the ligands is similar as reported method [40]. In a 100 mL sealed Schlenk tube, were placed 5,6,7-trihydroquinolin-8-one (1.47 g, 10.0 mmol), toluene (40 mL), 2,6-diisopropylaniline (1.77 g, 10.0 mmol), and *p*-toluenesulfonic acid hydrate (20 mg). The mixture was stirred overnight at 110 °C. Next, the mixture was cooled down to room temperature and filtered. The filtrate was dried under reduced pressure. The residue was dissolved in methanol and CH_2_Cl_2_ (*v*/*v* = 1/1). To this solution was added sodium borohydride (NaBH_4_, 3.78 g, 100 mmol) slowly, and the mixture was stirred overnight at room temperature. Water (50 mL) was added to quench the reaction. The product was extracted by chloroform and purified by column chromatography (silica gel, petroleum eather/ethyl acetate = 2/1) to be a yellow solid (1.31 g, 5.60 mmol, 56.0%). ^1^H NMR (CDCl_3_): δ 8.48 (d, 1 H, *J* = 4.5 Hz, quino–H), 7.42 (d, 1 H, *J* = 7.6 Hz, quino–H), 7.14–7.11 (m, 4 H, quino–H + Ar–H), 4.46 (br, 1 H, N–H), 4.04 (dd, 1 H, *J* = 8.5, 4.6 Hz, NCH), 3.59 (hept, 2 H, *J* = 6.9 Hz, C*H*Me_2_), 2.91–2.73 (m, 2 H, quino–H), 2.03–1.87 (m, 2 H, quino–H), 1.81–1.65 (m, 2 H, quino–H), 1.26 (d, 6 H, *J* = 6.9 Hz, CH(CH_3_)_2_), 1.18 (d, 6 H, *J* = 6.9 Hz, CH(CH_3_)_2_). ^13^C NMR (CDCl_3_): δ 157.37, 147.13, 145.65, 141.96, 136.92, 132.05, 124.76, 123.54, 122.08, 60.50, 29.27, 28.88, 27.56, 24.83, 24.26, 20.28. FT-IR (KBr, cm^−1^): 3311, 3060, 2955, 2864, 1574, 1457, 1417, 1324, 1250, 1194, 1147, 1104, 1055, 1002, 935, 807, 781, 745, 707, 546. Anal. Calcd for C_21_H_28_N_2_: C, 81.77; H, 9.15; N, 9.08. Found: C, 81.95; H, 9.01; N, 8.97.

*Synthesis of 8-(2,6-Me-anilide)-5,6,7-trihydroquinoline (**LH2**).* Using the method described above, 8-(2,6-Me-anilide)-5,6,7-trihydroquinoline was obtained as a yellow solid (1.28 g, 5.08 mmol, 50.8%) ^1^H NMR (CDCl_3_): δ 8.47 (d, 1 H, *J* = 4.3 Hz, quino–H), 7.42 (d, 1 H, *J* = 7.6 Hz, quino–H), 7.12 (dd, 1 H, *J* = 7.6, 4.7 Hz, quino–H), 7.02 (d, 2 H, *J* = 7.4 Hz, Ar–H), 6.87 (t, 1 H, *J* = 7.4 Hz, Ar–H), 4.37–4.36 (m, 1 H, NCH), 4.01 (br, 1 H, NH), 2.94–2.72 (m, 2 H, quino–H), 2.33 (s, 6 H, Me), 2.03–1.86 (m, 2 H, quino–H), 1.85–1.71 (m, 2 H, quino–H). ^13^C NMR (CDCl_3_): δ 157.68, 147.29, 145.05, 136.92, 132.08, 131.23, 128.79, 122.24, 122.09, 57.31, 29.76, 28.72, 19.57, 19.11. FT-IR (KBr, cm^–1^): 3333, 3042, 2940, 1589, 1570, 1471, 1438, 1256, 1214, 1186, 1160, 1093, 1033, 1013, 877, 846, 791, 752, 705, 681, 571. Anal. Calcd for C_17_H_20_N_2_: C, 80.91; H, 7.99; N, 11.10. Found: C, 80.78; H, 8.15; N, 11.02.

*Synthesis of 8-anilide-5,6,7-trihydroquinoline (**LH3**).* Using the method described above, 8-anilide-5,6,7-trihydroquinoline was obtained as a yellow solid (1.01 g, 4.51 mmol, 45.1%). ^1^H NMR (CDCl_3_): δ 8.45 (d, 1 H, *J* = 3.9 Hz, quino–H), 7.44 (d, 1 H, *J* = 7.6 Hz, quino–H), 7.21 (t, 2 H, *J* = 7.9 Hz, Ar–H), 7.17–7.10 (m, 1 H, quino–H), 6.78 (d, 2 H, *J* = 8.3 Hz, Ar–H), 6.73 (t, 1 H, *J* = 7.3 Hz, Ar–H), 4.86 (br, 1 H, NH), 4.50 (t, 1 H, *J* = 5.5 Hz, NCH), 2.94–2.75 (m, 2 H, quino–H), 2.35–2.30 (m, 2 H, quino–H), 2.01–1.84 (m, 2 H, quino–H). ^13^C NMR (CDCl_3_): δ 156.61, 148.16, 147.25, 137.14, 132.87, 129.28, 122.38, 117.63, 113.90, 54.04, 29.18, 28.56, 19.36. FT-IR (KBr, cm^−1^): 3320, 3096, 3054, 3016, 2943, 2865, 1604, 1516, 1441, 1312, 1255, 1160, 1108, 1021, 983, 864, 793, 737, 689, 508. Anal. Calcd for C_15_H_16_N_2_: C, 80.32; H, 7.19; N, 12.49. Found: C, 80.53; H, 7.18; N, 12.26.

*Synthesis of 8-(2,6-Me-4-Me-anilide)-5,6,7-trihydroquinoline (**LH4**).* Using the method described above, 8-(2,6-Me-4-Me-anilide)-5,6,7-trihydroquinoline was obtained as a yellow solid (1.67 g, 6.28 mmol, 62.8%). ^1^H NMR (CDCl_3_): δ 8.46 (d, 1 H, *J* = 4.3 Hz, quino–H), 7.41 (d, 1 H, *J* = 7.6 Hz, quino–H), 7.11 (dd, 1 H, *J* = 7.6, 4.7 Hz, quino–H), 6.85 (s, 2 H, Ar–H), 4.30–4.21 (m, 1 H, NCH), 3.98 (br, 1 H, NH), 2.94–2.69 (m, 2 H, quino–H), 2.30 (s, 6 H, Me), 2.25 (s, 3 H, Me), 2.00–1.87 (m, 2 H, quino–H), 1.84–1.71 (m, 2 H, quino–H). ^13^C NMR (CDCl_3_): δ 157.70, 147.28, 142.42, 136.98, 132.13, 131.95, 129.51, 122.21, 122.06, 57.82, 29.70, 28.82, 20.80, 19.68, 18.99. FT-IR (KBr, cm^–1^): 3326, 2936, 1570, 1483, 1439, 1368, 1299, 1228, 1156, 1088, 1014, 967, 849, 786, 736, 690, 579. Anal. Calcd for C_18_H_22_N_2_: C, 81.16; H, 8.32; N, 10.52. Found: C, 81.20; H, 8.21; N, 10.58.

### 2.3. Synthesis of Aluminum Complexes (**Al1**–**Al5**)

*Synthesis of **Al1**.* To a stirred solution of 8-(2,6-*^i^Pr*-anilide)-5,6,7-trihydroquinoline (0.308 g, 1.00 mmol) in dried toluene (30 mL) at room temperature, AlMe_3_ (1.00 mmol, 1.0 mL, 1 M in toluene) was added by syringe. The mixture was stirred at room temperature for 12 h and a yellow solution was obtained. The residue, after removing the solvent under vacuum, was washed by cold *n*-hexane (10 mL) to give a yellow powder (0.335 g, 0.92 mmol, yield 92%). ^1^H NMR (C_6_D_6_): δ 7.58 (d, 1 H, *J* = 5.1 Hz, quino–H), 7.30–7.22 (m, 3 H, quino–H + Ar–H), 6.59 (d, 1 H, *J* = 7.6 Hz, Ar–H), 6.35 (t, 1 H, *J* = 7.5 Hz, Ar–H), 4.48 (dd, 1 H, *J* = 11.6, 4.4 Hz, NCH), 4.17 (hept, 1 H, *J* = 6.8 Hz, C*H*Me_2_), 3.70 (hept, 1 H, *J* = 6.8 Hz, C*H*Me_2_), 2.17 (dd, 1 H, *J* = 17.5, 5.8 Hz, quino–H), 2.05 (dt, 1 H, *J* = 17.5, 8.7 Hz, quino–H), 1.86–1.77 (m, 1 H, quino–H), 1.42 (d, 3 H, *J* = 6.7 Hz, CH(C*H*_3_)_2_), 1.39 (d, 3 H, *J* = 6.7 Hz, CH(C*H*_3_)_2_), 1.37–1.32 (m, 2 H, quino–H), 1.30 (d, 3 H, *J* = 6.9 Hz, CH(C*H*_3_)_2_), 1.27 (d, 3 H, *J* = 6.9 Hz, CH(C*H*_3_)_2_), 1.25 (qd, 1 H, *J* = 12.2, 4.4 Hz, quino–H), −0.22 (s, 3 H, Al–Me), –0.25 (s, 3 H, Al–Me). ^13^C NMR (C_6_D_6_): δ 163.05, 149.62, 148.61, 144.06, 141.34, 139.11, 133.77, 129.23, 124.53, 124.26, 123.93, 122.80, 63.85, 29.32, 28.35, 26.98, 26.59, 26.22, 25.95, 25.59, 24.95, 20.52, −6.95, −8.59. Anal. Calcd for C_23_H_33_AlN_2_: C, 75.79; H, 9.13; N, 7.69. Found: C, 75.53; H, 8.98; N, 7.39.

*Synthesis of **Al2**.* Using the method described for **Al1**, **Al2** was obtained as a yellow powder (0.292 g, 0.95 mmol, yield 95%). ^1^H NMR (C_6_D_6_): δ 7.64 (d, 1 H, *J* = 5.2 Hz, quino–H), 7.35 (d, 1 H, *J* = 7.4 Hz, quino–H), 7.30 (d, 1 H, *J* = 7.3, Ar–H), 7.17 (t, 1 H, *J* = 7.5 Hz, quino–H), 6.68 (d, 1 H, *J* = 7.7 Hz, Ar–H), 6.43 (dd, 1 H, *J* = 7.5, 5.5 Hz, Ar–H), 4.58 (dd, 1 H, J = 11.6, 4.8 Hz, NCH), 2.65 (s, 3 H, Ar–Me), 2.59 (s, 3 H, Ar–Me), 2.27–2.18 (m, 1 H, quino–H), 2.14–2.07 (m, 1 H, quino–H), 1.88–1.83 (m, 1 H, quino–H), 1.44–1.26 (m, 2 H, quino–H), 1.13–1.05 (m, 1 H, quino–H), −0.14 (s, 3 H, Al–Me), −0.15 (s, 3 H, Al–Me). ^13^C NMR (C_6_D_6_): δ 163.34, 148.04, 141.35, 139.11, 138.52, 137.40, 133.84, 129.06, 128.62, 123.18, 122.67, 61.11, 29.06, 26.57, 20.60, 20.20, 19.85, −6.99, −8.31. Anal. Calcd for C_19_H_25_AlN_2_: C, 74.00; H, 8.17; N, 9.08. Found: C, 73.88; H, 8.01; N, 9.03.

*Synthesis of **Al3**.* Using the method described for **Al1**, **Al3** was obtained as a yellow powder (0.254 g, 0.91 mmol, yield 91%). ^1^H NMR (C_6_D_6_): δ 7.47 (br, 1 H, quino–H), 7.32 (br, 2 H, *J* = 7.6 Hz, Ar–H), 7.00 (d, 2 H, *J* = 7.5 Hz, Ar–H), 6.79 (t, 1 H, *J* = 7.0 Hz, Ar–H), 6.64 (d, 1 H, *J* = 7.3 Hz, quino–H), 6.39 (br, 1 H, quino–H), 4.24 (br, 1 H, NCH), 2.69 (br, 1 H, quino–H), 2.19–1.92 (m, 2 H, quino–H), 1.41–1.28 (m, 2 H, quino–H), 0.88-0.64 (m, 1 H, quino–H), −0.13 (s, 3 H, Al–Me), -0.19 (s, 3 H, Al–Me). ^13^C NMR (C_6_D_6_): δ 162.70, 152.63, 140.86, 139.31, 134.96, 129.76, 129.34, 123.19, 115.73, 57.21, 26.62, 25.81, 19.26, −6.82, −10.10. Anal. Calcd for C_17_H_21_AlN_2_: C, 72.83; H, 7.55; N, 9.99. Found: C, 72.55; H, 7.41; N, 9.67.

*Synthesis of **Al4**.* Using the method described for **Al1**, **Al4** was obtained as a yellow powder (0.309 g, 0.96 mmol, yield 96%). ^1^H NMR (C_6_D_6_): δ 7.58 (d, 1 H, *J* = 5.1 Hz, quino–H), 7.04 (s, 1 H, Ar–H), 6.99 (s, 1 H, Ar–H), 6.64 (d, 1 H, *J* = 7.5 Hz, quino–H), 6.37 (dd, 1 H, *J* = 7.4, 5.6 Hz, quino–H), 4.50 (dd, 1 H, *J* = 11.6, 4.7 Hz, NCH), 2.52 (s, 3 H, Ar–Me), 2.47 (s, 3 H, Ar–Me), 2.29 (s, 3 H, Ar–Me), 2.22–2.14 (m, 1 H, quino–H), 2.09–2.02 (m, 1 H, quino–H), 1.84–1.79 (m, 1 H, quino–H), 1.37–1.22 (m, 2 H, quino–H), 1.07–0.99 (m, 1 H, quino–H), −0.23 (s, 3 H, Al–Me), −0.26 (s, 3 H, Al–Me). ^13^C NMR (C_6_D_6_): δ 163.58, 145.02, 141.37, 139.04, 138.14, 137.04, 133.88, 131.72, 129.83, 129.41, 122.64, 61.32, 29.08, 26.61, 21.04, 20.48, 20.24, 19.71, –7.05, –8.37. Anal. Calcd for C_20_H_27_AlN_2_: C, 74.50; H, 8.44; N, 8.69. Found: C, 74.22; H, 8.37; N, 8.51.

*Synthesis of **Al5**.* Using the method described for **Al1**, **Al5** was obtained as a yellow powder (0.325 g, 0.93 mmol, yield 93%). ^1^H NMR (C_6_D_6_): δ 7.72 (d, 1 H, *J* = 5.0 Hz, quino–H), 7.02 (s, 1 H, Ar–H), 6.97 (s, 1 H, Ar–H), 6.67 (d, 1 H, *J* = 7.6 Hz, quino–H), 6.42 (dd, 1 H, *J* = 7.2, 5.7 Hz, quino–H), 4.49 (dd, 1 H, *J* = 11.4, 4.6 Hz, NCH), 2.52 (s, 3 H, Ar–Me), 2.47 (s, 3 H, Ar–Me), 2.27 (s, 3 H, Ar–Me), 2.21–2.14 (m, 1 H, quino–H), 2.10–2.03 (m, 1 H, quino–H), 1.85–1.80 (m, 1 H, quino–H), 1.42 (t, 3 H, *J* = 8.1 Hz, CH_2_C*H*_3_), 1.36–1.31 (m, 2 H, quino–H), 1.27 (t, 3 H, *J* = 8.1 Hz, CH_2_C*H*_3_), 1.05 (qd, 1 H, *J* = 12.0, 5.0 Hz, quino–H), 0.48–0.31 (m, 4 H, C*H*_2_CH_3_). ^13^C NMR (C_6_D_6_): δ 163.88, 145.28, 141.58, 139.11, 137.99, 136.82, 134.13, 131.69, 129.86, 129.40, 122.56, 61.79, 29.33, 26.56, 21.02, 20.35, 20.18, 19.56, 10.49, 9.94, 1.98, 1.21. Anal. Calcd for C_22_H_31_AlN_2_: C, 75.39; H, 8.92; N, 7.99. Found: C, 75.13; H, 8.78; N, 7.95.

### 2.4. The ROP of ε-CL and rac-LA

A general procedure for homopolymerization in the presence of benzyl alcohol (run 3, Table 1) is given as follows and other ROPs of ε-CL and *rac*-LA including the copolymerization were carried out by the similar procedure described here. A toluene solution of **Al2** (0.020 mmol), BnOH (0.020 mmol), and ε-caprolactone (5.0 mmol), along with 3.44 mL toluene, was added into a Schlenk tube at room temperature in glove-box. The tube was taken out and placed into the oil bath at 110 °C for the 30 min. Then, the mixture was quenched by few drops of glacial acetic acid. A little amount of solution was transferred to another Schlenk tube, and all the volatiles were removed under vacuum. The residues were dissolved in CDCl_3_ for ^1^H NMR characterization to determine the conversion. The rest solution was poured into methanol (200 mL) to precipitate the polymer. The resultant polymer was then collected by filtration and dried in vacuo.

### 2.5. Crystal Structure Determinations

Single crystals of **Al4** and **Al5** were grown by diffusion of *n*-hexane into their toluene solutions slowly at room temperature. X-ray diffractions for **Al4** and **Al5** were carried out at 173(2) K on a Rigaku RAXIS Rapid IP diffractometer with graphite-monochromated Mo Kα radiation (λ = 0.71073 Å). The structures were solved with the method of XS [42] and refined with ShelXL [43] according to Olex2 [44]. The hydrogen atoms were calculated and introduced riding on the corresponding parent atoms. Crystal data for **Al4** and **Al5** were summarized in Table S1 in ESI. CCDC reference numbers 1495111 and 1495112 were for complexes **Al4** and **Al5**, respectively.

## 3. Results and Discussion

### 3.1. Synthesis and Characterization of the Ligands and Complexes

**Scheme 2:**
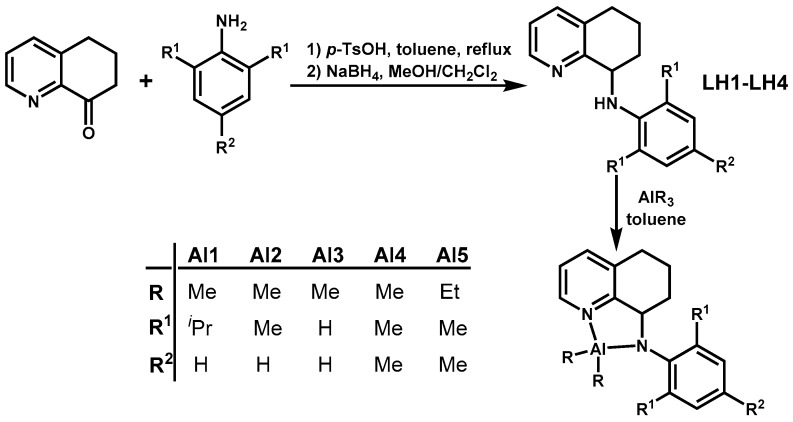
Synthesis of the Ligands (**LH1**–**LH4**) and Al complexes (**Al1**–**Al5**).

The 8-substituted-anilide-5,6,7-trihydroquinoline ligands (Scheme 2, **LH1**-**LH4**) were prepared using the modified procedure reported previously [40]. The ligands **LH1**-**LH4** were prepared by reduction of the imine analogue with NaBH_4_ in the mixture of MeOH and CH_2_Cl_2_ (*v*/*v* = 1/1), which accounts for faster reaction and higher yields (45%–63%). The Al complexes **Al1**–**Al5** (Scheme 2) were synthesized as yellow solids by the stoichiometric reactions of AlMe_3_ or AlEt_3_ and the corresponding ligands in toluene overnight at room temperature in high yields (91%–96%). **Al1**–**Al5** are highly sensitive to air and moisture. However, they can be conserved without decomposition over months under N_2_ or in the glove-box.

**Figure 1:**
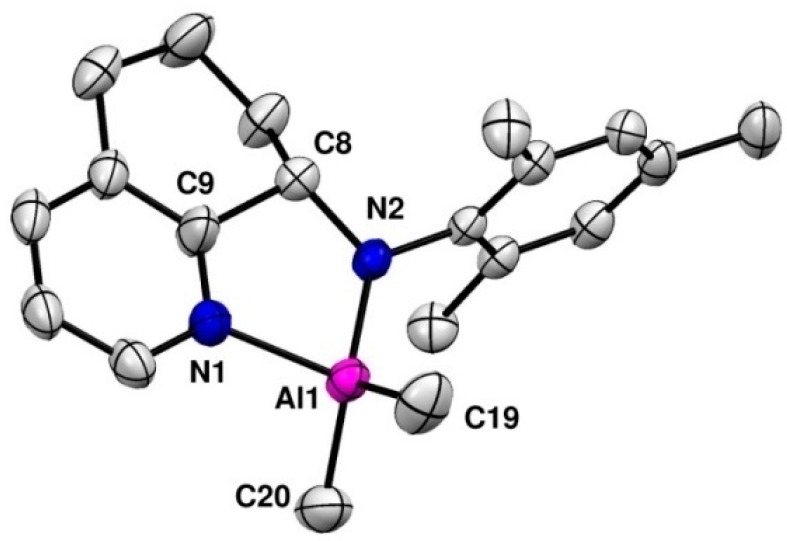
ORTEP of the molecular structure of **Al4**. Ellipsoids at 50% probability level. Hydrogen atoms are omitted for clarity. Selected distances (Å) and angles (deg): A1–N1 1.981(3), A1–N2 1.844(2), A1–C19 1.971(3), A1–C20 1.972(4); N1–Al1–N2 85.07(10), N1–Al1–C20 105.08(14), N1–Al1–C19 111.83(14), N2–Al1–C19 117.26(14), N2–Al1–C20 120.84(13), C19–Al1–C20 112.16(17).

**Figure 2:**
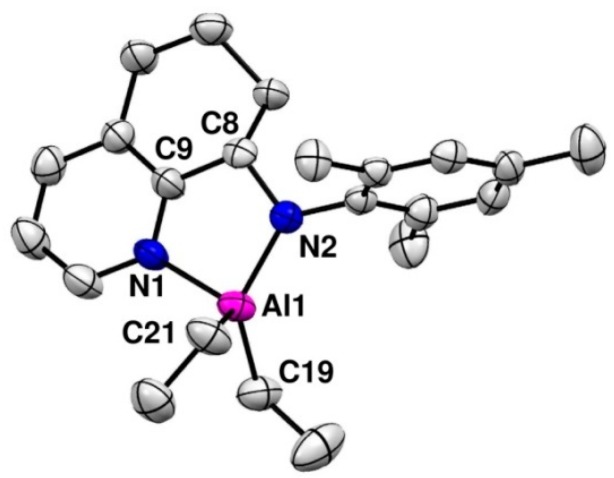
ORTEP of the molecular structure of **Al5**. Ellipsoids at 50% probability level. Hydrogen atoms are omitted for clarity. Selected distances (Å) and angles (deg): A1–N1 1.980(3), A1–N2 1.844(3), A1–C19 1.974(3), A1–C21 1.971(4); N1–Al1–N2 85.13(12), N1–Al1–C21 109.55(17), N1–Al1–C19 106.06(16), N2–Al1–C19 120.76(17), N2–Al1–C21 117.96(15), C19–Al1–C21 112.27(18).

Complexes **Al1**–**Al5** were characterized by NMR spectra (^1^H and ^13^C) and elemental analysis, which were consistent with the chemical structure of LAlR_2_. In the ^1^H NMR spectrum of **Al1**, as compared to that of the corresponding ligand **LH1**, the new additional resonances in the high field region (−0.22 to −0.25 ppm) were observed and attributed to the methyl groups on Al center (Al–C*H*_3_). In the meantime, the N–*H* signal (broad resonance at 4.46 ppm) of **LH1** disappeared as expected. In addition, there were two sets of resonances of *CH*(CH_3_)_2_ (4.17 and 3.70 ppm) for **Al1** (Figure S4 in ESI), which was distinguished from only one (3.59 ppm) for **LH1**. It was assumed that the aryl-N bonding of complex **Al1** could not freely rotate in solution because of the steric hindrance of the *ortho*-isopropyl groups of the *N*-aryl rings in Al complex. The similar characteristics were even observed for complexes **Al2**–**Al5** with less steric hindrance. The structures of **Al4** and **Al5** were determined by X-ray crystallography and depicted in Figure 1 and Figure 2 with the selected bond lengths and angles. In the molecule of **Al4**, the geometry around Al can be best described as a distorted tetrahedron, as evidenced in the bond angles for N1–Al1–N2 = 85.07(10), N1–Al1–C20= 105.08(14), N1–Al1–C19 = 111.83(14), N2–Al1–C19 = 117.26(14), N2–Al1–C20 = 120.84(13). The bond distance of Al1–N1 (1.981(3)) was significant longer than that of Al1–N2 (1.844(2)), indicating two different types of bonding. The aryl ring was almost perpendicular to the coordination plane with the dihedral angle of 78.19°. The coordination features (geometry and coordination mode) of complex **Al5** (Figure 2) were similar as those of complex **Al4**, despite the different alkyls (Me or Et) on Al centres.

### 3.2. Ring-opening Polymerization of ε-CL and rac-LA

Catalytic performances of complexes **Al1**–**Al5** for ε-CL homopolymerization were examined and the results were shown in Table 1. The catalytic system using **Al2** without alcohol produced PLAs with a broad dispersity Ð (Table 1 run 1), consistent with the fact that the metal alkoxides generally polymerized cyclic esters in better controllable way than their alkyl analogues [45,46,47,48,49]. We previously reported that quinolin-8-amine-Al complexes had high activity for ROP of ε-CLwithout the addition of alcohol, but being short of a controlled manner [45]. In contrast, Nomura reported that for phenoxy-imine-Al complexes, addition of alcohol was essential, and the polymerization did not occur in the absence of alcohol [1]. Thus, the polymerizations using other Al complexes (**Al1**, **Al3**–**Al5**) without an alcohol were not investigated further. Instead, benzyl alcohol, one equivalent to metal, was employed to generate in situ the aluminum benzyloxide, which can act as the catalyst via a coordination insertion mechanism. This was consistent with the analysis of ^1^H NMR of resultant polymer possessing a benzyl as the end group (see ESI, Figure S1). All catalytic systems (runs 2–6, Table 1) exhibited very high efficiency for ROP of ε-CL with the conversion of 99%–100% according to Redshaw’s classification of the activity for ROP of ε-CL [1], and produced PCLs with narrow dispersity Ð of 1.18–1.24, which was believed as a living/controlled polymerization process. The different ligands with different substituents on the aryl and the alkyl groups on the Al center had no clear distinctive influence on the catalytic performance in regarding to the activities.

**Figure 3:**
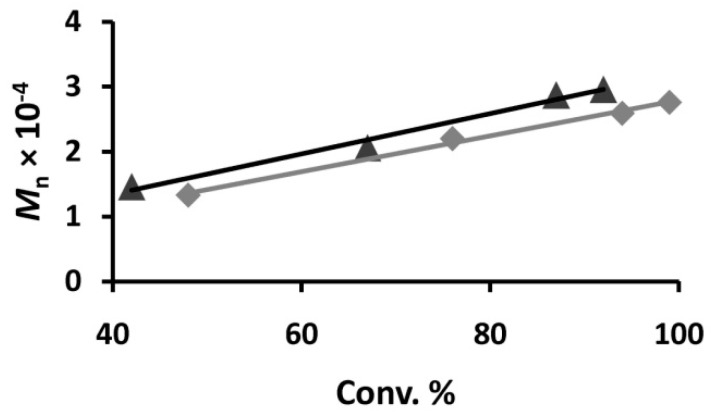
*M*_n_ vs. monomer conversion in the ROP of ε-CL (gray, runs 3 and 7–9 in Table 1) and *rac*-LA (dark, runs 2 and 6–8 in Table 2) initiated by **Al2**/BnOH.

**Figure 4:**
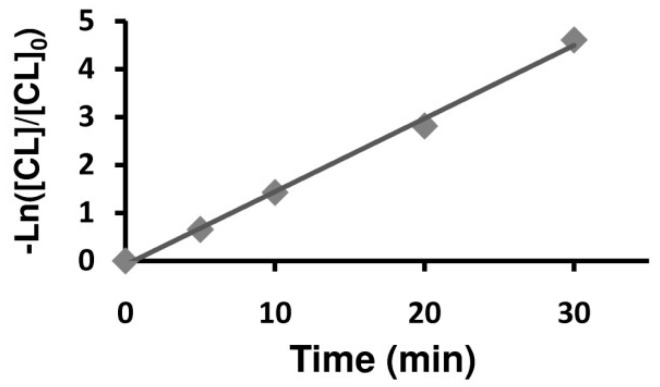
Plots of -Ln([CL]/[CL]_0_) vs. time in the ROP of ε-CL initiated by **Al2**/BnOH (runs 3 and 7–9, Table 1).

According to runs 3 and 7–9 in Table 1, the linear relationship between the conversion of monomer and *M*_n_ was observed, together with narrow dispersity Ð (1.10–1.18), suggesting a typical living polymerization process (Figure 3, gray). The dispersity Ð (*M*_w_/*M*_n_) of the produced polyesters were somewhat broad with increased monomer conversions, indicating sort of transesterification accompanied by the propagation. Similar phenomenon was also observed for quinolin-8-amine-Al system [45]. Note that from Figure 4 the rate of the ROPs was first-order dependent upon the monomer concentration, which was also observed for other systems [14,35]. This was agreement with a living polymerization process for the current catalytic systems. As observed for the catalytic system **Al2**/BnOH, increasing molar ratios of CL/Al (runs 3, 10 and 11, Table 1), led to higher molar mass polymers but less efficient. Note that increasing the amount of alcohol (molar ratio of BnOH/Al from 1 to 10, runs 3, 12, and 13, Table 1), the polymerization went quite well and the additional alcohol decreased the *M*_n_, while the dispersity Ð kept almost invariant (narrow and monomodel). According to ^1^H NMR of produced PCL (run 13, Table 1; Figure S1 in ESI), the *M*_n (NMR)_ (3000 g∙mol^−1^) could be excellent to match the values of *M*_n (GPC)_ (3100 g∙mol^−1^) and *M*_n (cal.)_ (2900 g∙mol^−1^). Take these into account, the current Al complexes were tolerant to excess of alcohol and thus a highly catalytic efficiency was achieved, which was recognized as immortal polymerization with the advantages of atom economy, molar masscontrol, and low metal residues [7,50,51,52,53,54,55].

The ROP of *rac*-LA by complexes **Al1**–**Al5** in the presence of BnOH were also investigated and the results were tabulated in Table 2. Compared to the ROP of ε-CL, it was obviously that under the identical conditions all catalytic systems showed less efficient for the ROP of *rac*-LA, similar as other reported catalytic systems [56,57]. However, within 12 h, high conversions (92%–96%) were obtained with narrow dispersity Ð. Moreover, a linear relationship was observed (Figure 3, dark) between the monomer conversions and the *M*_n_s, suggesting a controlled manner of polymerization. The decoupled ^1^H NMR spectra of PLAs (Figure S2 in ESI) indicated atactic PLAs obtained, which is in contrast to well-known stereoselective salen-Al catalytic system [29].

Copolymers of ε-CL and *rac*-LA, particularly the random copolymers, are intriguing biodegradable materials with improved properties as comparing to their homopolyesters.[30,58,59,60,61,62] Thus, copolymerization of ε-CL and *rac*-LA with **Al2**/BnOH (run 9, Table 2) was tested under similar polymerization conditions as homopolymerization. Unfortunately, the ^1^H NMR results showed that, as most of other reported systems, LA was far more preferentially polymerized as compared to CL during the copolymerization [57]. The analysis of the spectrum indicated an entire conversion of LA and 6.1% of CL (Figure S3 in ESI). Consequently, a gradient polymer rather than a random one was prepared in this case. As mentioned in the introduction, Nomura introduced a bulky group, *^i^*Pr_3_Si, on the *ortho*-phenoxy positions for the salen-Al system, resulting in the strict random copolymerization of *ε*-CL and LA for the very first time with a designed catalyst [30]. Nowadays we are also working on the modification of the structure of Al complexes to achieve copolymerization with better control.

## 4. Conclusions

Dialkylaluminum complexes (**Al1**–**Al5**) bearing 8-anilide-5,6,7-trihydroquinoline ligands were prepared and characterized by ^1^H and ^13^C NMR and elemental analysis. The solid state structures of **Al4** and **Al5** were analyzed by X-ray diffractions and revealed distorted tetrahedral geometries around Al centers. All the Al complexes were highly active toward the ring-opening homopolymerization of ε*-*CL and *rac*-LA in the presence of one equivalent of BnOH in a living/controlled manner. Note that the excess of alcohol to Al initiator would lead to an efficient catalytic system rather than termination of the active propagation processes, although the resultant polymers possessed low molar masss with narrow distributions.

## Figures and Tables

**Table 1:** Homopolymerization ROP of *ε*-CL by **Al1**–**Al5**/BnOH *^a^.*

run	Com.	CL:Al:BnOH	*t* min	conv. (%) *^b^*	*M*_n_ *^c^* × 10^−4^	Ð*^c^*	*M*_n_ *^d^*_(cal.)_ × 10^−4^
1	**Al2**	250:1:0	30	98	3.79	1.59	-
2	**Al1**	250:1:1	30	99	2.27	1.20	2.83
3	**Al2**	250:1:1	30	99	2.76	1.18	2.83
4	**Al3**	250:1:1	30	99	2.86	1.19	2.83
5	**Al4**	250:1:1	30	100	2.50	1.23	2.86
6	**Al5**	250:1:1	30	99	2.43	1.24	2.83
7	**Al2**	250:1:1	5	48	1.33	1.10	1.37
8	**Al2**	250:1:1	10	76	2.20	1.10	2.16
9	**Al2**	250:1:1	20	94	2.59	1.12	2.68
10	**Al2**	125:1:1	30	100	1.44	1.14	1.43
11	**Al2**	500:1:1	30	80	3.59	1.46	4.57
12	**Al2**	250:1:5	30	99	0.54	1.07	0.51
13	**Al2**	250:1:10	30	99	0.31	1.13	0.29

*^a^* Conditions: 20 μmol Al, 1.0 M ε*-*CL toluene solution, 110 °C. *^b^* Determined by ^1^H NMR. *^c^* GPC data in THF vs. polystyrene standards, using a correcting factor 0.56 [41]. *^d^ M*_n (cal.)_ = *M*_CL_ × ([CL]:[Al]) × ([Al]:[BnOH]) × conversion + *M*_BnOH_.

**Table 2:** Homopolymerization ROP of *rac*-LA by **Al1**–**Al5**/BnOH *^a^.*

run	Com.	*t*/h	conv. (%) *^b^*	*M*_n_ *^c^* × 10^−4^	Ð*^c^*	*M*_n_ *^d^* (cal.) × 10^−4^
1	**Al1**	12	96	2.65	1.31	3.46
2	**Al2**	12	96	2.96	1.26	3.33
3	**Al3**	12	94	2.34	1.38	3.39
4	**Al4**	12	95	3.02	1.32	3.42
5	**Al5**	12	96	2.89	1.36	3.46
6	**Al2**	1	42	1.46	1.12	1.52
7	**Al2**	3	67	2.06	1.15	2.42
8	**Al2**	6	87	2.87	1.32	3.13
9 *^e^*	**Al2**	12	100/6.1 *^f^*	3.58	1.05	3.78

*^a^* Conditions: 20 μmol Al, 1.0 M *rac*-LA toluene solution, [LA]:[Al]:[BnOH] = 250:1:1, 110 °C. *^b^* Determined by ^1^H NMR. *^c^* GPC data in THF vs. polystyrene standards, using a correcting factor 0.58 [41]. *^d^* *M*_n (cal.)_ = *M*_LA_ × ([LA]:[Al]) × ([Al]:[BnOH]) × conversion + *M*_BnOH_. *^e^* Copolymerization of ε*-*CL and *rac*-LA, [LA]:[CL]:[Al]:[BnOH] = 250:250:1:1. *^f^* conversions of LA/CL.

## Supplementary Materials

The following are available online at www.mdpi.com/2073-4360/9/3/83/s1, Electronic Supplementary Information (ESI) for NMR of polymers and **Al1** (Figure S1–S4) and crystal data and structure refinement (Table S1), Crystallographic details (CIFs of **Al4** and **Al5**).
