# Supplementary material for: Synthesis of Aluminum Complexes Bearing 8-Anilide-5,6,7-trihydroquinoline Ligands: Highly Active Catalyst Precursors for Ring-Opening Polymerization of Cyclic Esters
Source: Polymers (Basel). 2017 Mar 1;9(3):83. doi: 10.3390/polym9030083 (PMC6432074; doi:10.3390/polym9030083)
Supplement: Supplementary file 1 [file polymers-09-00083-s001.pdf]

# Supplementary Materials: Synthesis of Aluminum Complexes Bearing 8-Anilide-5,6,7-trihydroquinoline Ligands: Highly Active Catalyst Precursors for Ring-opening Polymerization of Cyclic Esters

**Shaofeng Liu, Jie Zhang, Weiwei Zuo, Wenjuan Zhang, Wen-Hua Sun, Hongqi Ye and Zhibo Li**

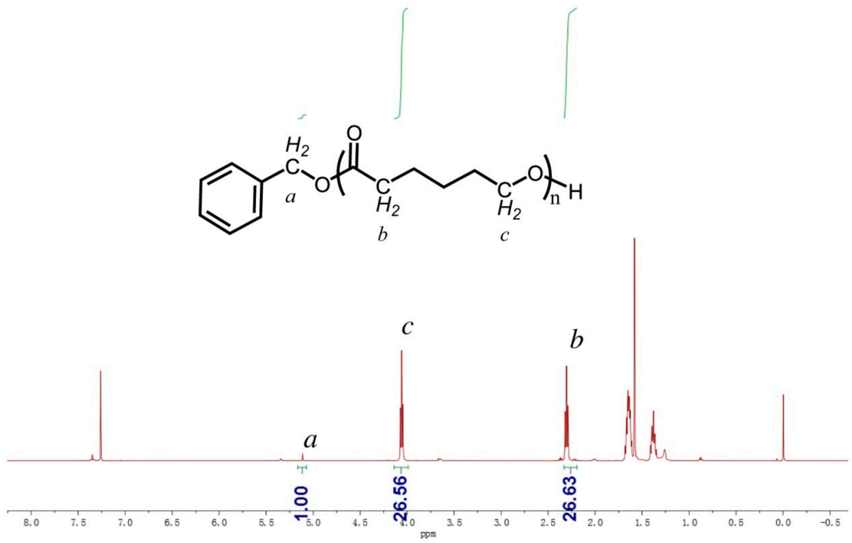


**Figure S1.** $^{1}$H NMR of PCL in $CDCl_3$ obtained by **Al2** in the presence of 10 equiv. of BnOH (entry 13, Table 1).

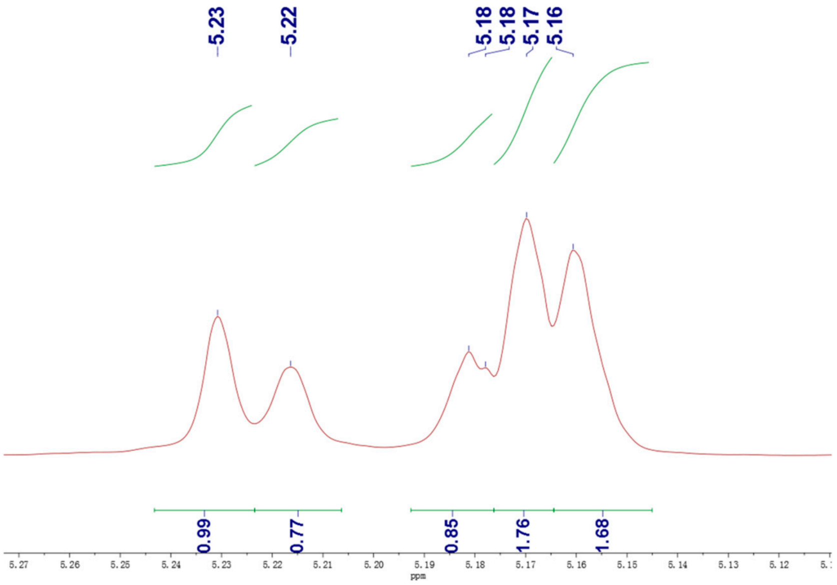


**Figure S2.** Homonuclear decoupled $^{1}$H NMR in $CDCl_3$ spectrum of the methine region of PLA prepared with **Al2**/BnOH (entry 2 Table 2).

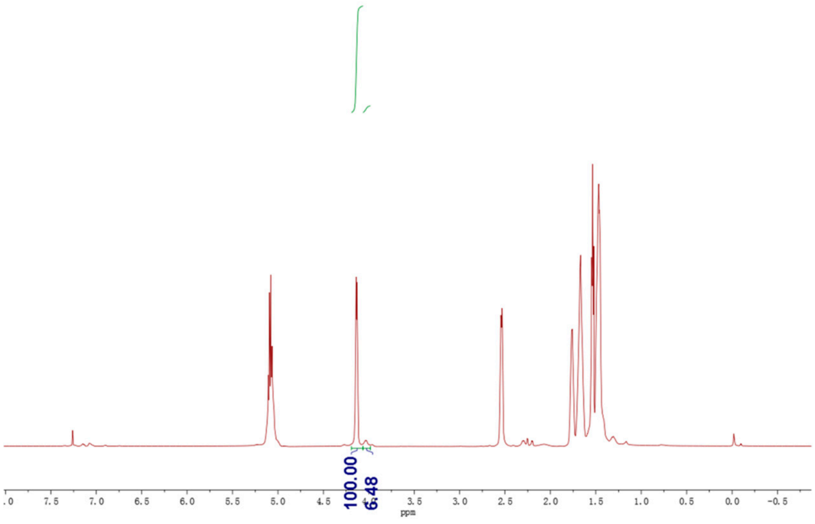

**Figure S3.** $^1H$ NMR of copolymer of P(LA-*co*-CL) in $CDCl_3$ obtained by **Al2/**BnOH (entry 9, Table 2).

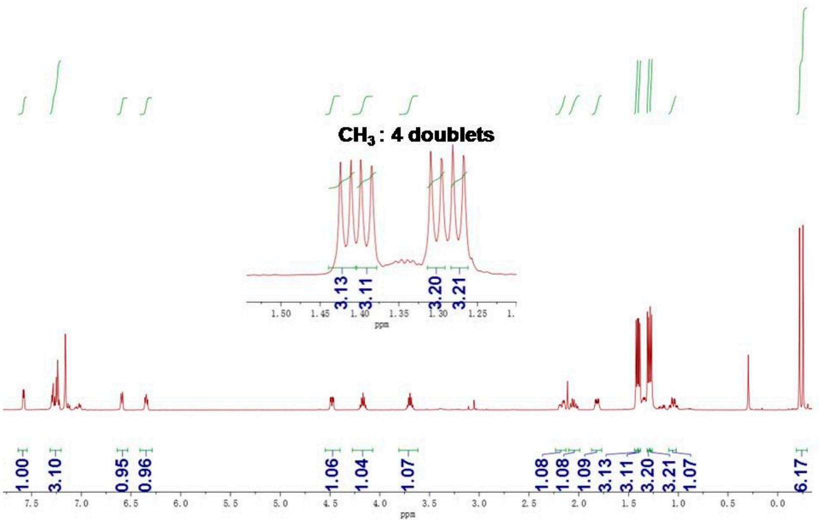


**Figure S4.** $^1H$ NMR of **Al1**.

**Table S1.** Crystal data and structure refinement for **Al4** and **Al5**.

| Complex | Al4 | Al5 |
| --- | --- | --- |
| Empirical formula | $C_{20}H_{27}AlN_2$ | $C_{22}H_{31}AlN_2$ |
| Formula weight | 322.41 | 350.47 |
| Temperature / K | 173.15 | 173.15 |
| Crystal system | Orthorhombic | Trigonal |
| Space group | $P2_12_12_1$ | R-3 |
| a / Å, b / Å, c / Å | 9.5699(6)<br>11.8000(9)<br>16.6399(11) | 37.075(12)<br>37.075(12)<br>7.841(2) |
| α/°, β/°, γ/° | 90, 90, 90 | 90, 90, 120 |
| Volume / Å$^3$ | 1879.1(2) | 9334(7) |
| Z | 4 | 18 |
| $\rho_{calc}$ / mg mm$^{-3}$ | 1.140 | 1.122 |
| μ / mm$^{-1}$ | 0.110 | 0.104 |
| F(000) | 696 | 3420 |
| Crystal size / mm$^3$ | 0.438 × 0.247 × 0.122 | 0.295 × 0.239 × 0.176 |
| 2Θ range for data collection | 2.996 to 27.464° | 2.197 to 25.197° |
| Index ranges | -12<=h<=12<br>-15<=k<=13<br>-21<=l<=18 | -44<=h<=27<br>-42<=k<=44<br>9<=l<=8 |
| Reflections collected | 13458 | 11893 |
| Independent reflections | 4290 [R(int) = 0.0397] | 3728 [R(int) = 0.0586] |
| Data/restraints/parameters | 4290/0 /232 | 3728/12/259 |
| Goodness-of-fit on F$^2$ | 1.117 | 1.183 |
| Final R indexes [I>2σ (I)] | $R_1$ = 0.0485<br>$wR_2$ = 0.1098 | $R_1$ = 0.0902<br>$wR_2$ = 0.1659 |
| Final R indexes [all data] | $R_1$ = 0.0513<br>$wR_2$ = 0.1115 | $R_1$ = 0.1170<br>$wR_2$ = 0.1803 |
| Largest diff. peak/hole/e Å$^{-3}$ | 0.197/-0.162 | 0.303/-0.243 |
